# Panda profiles: integrating personality, cognition, and physiology for conservation success

**DOI:** 10.1093/conphys/coag019

**Published:** 2026-03-27

**Authors:** Yuhang Li, Cong Yu, Jing Liao, Yuxin Jiang, Tianjiao Liu, Lijun Luo, Shuai Yang, Bo Yang, Xiang Xu, Tao Deng, Desheng Li, Jianghong Ran

**Affiliations:** Sichuan Key Laboratory of Conservation Biology on Endangered Wildlife, College of Life Sciences, Sichuan University, Chengdu, Sichuan 610065, China; Laboratory of Wildlife Ecology and Behaviour, School of Biology and Environmental Science, University College Dublin, Dublin 4, D04F5K2, Ireland; Sichuan Key Laboratory of Conservation Biology on Endangered Wildlife, College of Life Sciences, Sichuan University, Chengdu, Sichuan 610065, China; Sichuan Key Laboratory of Conservation Biology on Endangered Wildlife, College of Life Sciences, Sichuan University, Chengdu, Sichuan 610065, China; Sichuan Key Laboratory of Conservation Biology on Endangered Wildlife, College of Life Sciences, Sichuan University, Chengdu, Sichuan 610065, China; Sichuan Key Laboratory of Conservation Biology on Endangered Wildlife, College of Life Sciences, Sichuan University, Chengdu, Sichuan 610065, China; China Conservation and Research Centre for the Giant Panda, Shuxin Road, Qingchengshan Town, Dujiangyan, Chengdu, Sichuan 610081, China; China Conservation and Research Centre for the Giant Panda, Shuxin Road, Qingchengshan Town, Dujiangyan, Chengdu, Sichuan 610081, China; China Conservation and Research Centre for the Giant Panda, Shuxin Road, Qingchengshan Town, Dujiangyan, Chengdu, Sichuan 610081, China; China Conservation and Research Centre for the Giant Panda, Shuxin Road, Qingchengshan Town, Dujiangyan, Chengdu, Sichuan 610081, China; China Conservation and Research Centre for the Giant Panda, Shuxin Road, Qingchengshan Town, Dujiangyan, Chengdu, Sichuan 610081, China; Key Laboratory of SFGA on The Giant PandaNo. 19, South Section 4, Yihuan Road, Wuhou District, Chengdu, Sichuan 610081, China; Sichuan Key Laboratory of Conservation Biology on Endangered Wildlife, College of Life Sciences, Sichuan University, Chengdu, Sichuan 610065, China

**Keywords:** Behavioural syndromes, conservation strategies, FGCM concentrations, lateralization

## Abstract

The alarming decline in biodiversity necessitates a comprehensive understanding of animal adaptations to environmental changes. Behavioural syndromes, which encompass consistent patterns of behaviour across various contexts, play a crucial role in animal adaptation and survival. In this study, we investigated behavioural syndromes in giant pandas (*Ailuropoda melanoleuca*), a keystone species in global conservation efforts. Using a comprehensive array of behavioural assays, we assessed the cognitive functions and personality traits of giant pandas, linking these traits to physiological stress indicators—faecal glucocorticoid metabolite (fGCM) concentrations—and established behavioural syndromes across both captive and wild settings. Our findings delineate proactive-reactive coping styles, demonstrating that proactive individuals exhibit lower baseline fGCM concentrations and enhanced exploratory behaviours, thereby suggesting superior stress resilience. Additionally, we identified clear lateralization in behaviour and found that left-handedness and stronger lateralization were indicative of a more reactive personality, underscoring the functional differences between brain hemispheres in controlling emotions and behaviours. Field studies extended these insights to wild populations, suggesting that fGCM concentrations may exhibit contrasting relationships with human disturbance at intra-individual and inter-individual levels in pandas. This study is the first to integrate personality traits, cognitive abilities, and hormonal baselines in giant pandas. Building on these integrative findings, we propose a conservation framework that embeds individual behavioural phenotypes into management decisions and calls for customized reintroduction and protection strategies.

## Introduction

In the context of rapid declines in biodiversity and ecosystem functions (Watson *et al.*, 2019), understanding how animals adapt to environmental changes has become a central issue in global scientific research. Animal behaviour is critical in species' adaptation to anthropogenic changes, helping elucidate why some species thrive in human-altered environments while others decline (Sih *et al.*, 2011; Candolin and Wong, 2012; Sih, 2013). As research on animal behaviour evolves, there has been a shift from understanding at the species and population levels (Tinbergen, 2005) to a focus on individual differences (Dall *et al.*, 2004), aiming to understand the detailed ecological and evolutionary causes and consequences of individual variability (Réale *et al.*, 2007b; Wolf and Weissing, 2012). The immediate reactions of individuals to their environments substantially determine their survival conditions—how individuals successfully navigate challenges and changes affects their dispersal and migration patterns, movement behaviours, habitat choices, foraging strategies, predator avoidance, mating tactics and social behaviours, ultimately influencing the ecology and evolution of populations and species (Wcislo, 1989; Biro and Stamps, 2008; Duckworth, 2009; Wolf and Weissing, 2012; Moiron *et al.*, 2020).

Adaptive responses exhibited by individuals, which encompass their behavioural patterns, cognitive abilities (Shettleworth, 2009), and underlying neuroendocrine adjustments (Houslay *et al.*, 2018), are not only products of adaptive evolution but also key drivers of dynamic ecological processes (Sih *et al.*, 2012). These multifaceted responses show considerable inter-individual variability. Within this variability, animal personality is defined as consistent between-individual differences in behaviour that are stable across time and various ecological contexts (Réale *et al.*, 2007a; Stamps and Groothuis, 2009). Often, specific personality traits are correlated, forming what are known as behavioural syndromes—suites of inter-correlated behaviours expressed either within a given behavioural context or across different contexts (Sih *et al.* 2004). The expression of these correlated behaviours is often intrinsically linked to, and can reflect underlying individual differences in cognitive styles or processes (Carere and Locurto, 2011; Sih and Del Giudice, 2012), such as variations in learning, memory or decision-making.

When faced with adverse or uncontrollable environmental challenges, animals deploy an integrated stress response, drawing on both behavioural adjustments and physiological mechanisms to maintain homeostasis (Selye, 1973; Johnson *et al.*, 1992; Chrousos, 1998); glucocorticoids, such as cortisol, are central hormonal mediators of this process (Øverli *et al.*, 2007; Carere *et al.*, 2010). While growing evidence shows that behavioural syndromes often parallel variation in cortisol (Carere and Van Oers, 2004; Cockrem, 2007; Koolhaas *et al.*, 2010), other studies report weak or inconsistent links (Qu *et al.*, 2018; Westrick *et al.*, 2019), underscoring ecological and phylogenetic contingencies (Korte *et al.*, 2005; Biro and Stamps, 2008). This variability in correlations highlights the critical importance of species-specific and context-dependent studies. Incorporating personality and cognition into experimental biology not only advances our understanding of trait evolution and ecology but also aids in predicting, maintaining and adjusting population responses to environmental changes, thus guiding species conservation, especially for rare and wild animals (Wolf and Weissing, 2012; Roche *et al.*, 2016).

The giant panda (*Ailuropoda melanoleuca*), endemic to China, is a global conservation icon and umbrella species (Swaisgood *et al.*, 2016). Research on this species is crucial not only for the direct conservation of pandas but also for the protection of coexisting species and broader biodiversity conservation (Shen *et al.*, 2023). Surprisingly, studies on giant panda personality and cognitive abilities are relatively sparse. Among these limited studies, Martin-Wintle *et al.* (2017) explored the impact of personality on reproductive success, and Perdue *et al.* (2011) identified sex differences in spatial abilities among pandas. Ma *et al.* (2015) and Li *et al.* (2017) focused on cognitive abilities, noting facial recognition capabilities to a certain point but no self-recognition. Deng *et al.* (2014) linked faecal glucocorticoid metabolite (fGCM) concentrations to human disturbance pressures. However, there is a noticeable gap in combining cognition, personality and hormones together, underscoring the urgent need for deeper behavioural insights into giant pandas, which could not only optimize conservation strategies but also provide new perspectives for conservation theory and practice. Moreover, given the extinction risk faced by pandas due to significant habitat loss and reduced range (Lenoir and Svenning, 2014), and the Chinese government’s reintroduction plans (Wei *et al.*, 2015a, 2015b; Huang, 2016; Yang *et al.*, 2018; Wang, 2019), it becomes essential to quantify individual personalities before wild releases, as personality is linked to animal dispersal behaviours (Biro and Stamps, 2008; Smith and Blumstein, 2008; Merrick and Koprowski, 2017; Spiegel *et al.*, 2017; Kelleher *et al.*, 2018; West *et al.*, 2019). This approach can substantially enhance the success rates of reintroduction and translocation projects, which are inherently challenging (Wolf *et al.*, 1998; Fischer and Lindenmayer, 2000; Resende *et al.*, 2020).

Our study is the first to establish interconnections among the personality, cognitive abilities, handedness and faecal hormone levels of giant pandas. Initially, we utilized three experimental approaches—the Multi-Access Box (MAB) paradigm, Resource Depletion Test and String-Pulling Task—to quantify cognitive performances, personality differences and handedness among captive giant pandas, exploring whether their individual personalities, cognitive abilities, handedness and endocrine levels integrate in a manner consistent with behavioural syndrome models. Moreover, using geographic distribution data and faecal hormone levels from wild individuals, we employed novel assessment methods to detect coping styles in the wild, proposing for the first time a theoretical framework for reintroducing giant pandas that underscores the importance of considering personality in panda conservation. This comprehensive approach not only reveals the complex interplay between individual behaviour and physiological response but also underscores the critical role of individual differences in species adaptability and survival strategies. Specifically, we hypothesized that physiological profiles (as a proxy for personality) would correlate with spatial distribution in the wild, predicting that proactive individuals (characterized by lower constitutive fGCM) would exhibit greater tolerance to anthropogenic disturbance and thus be found at shorter distances to human activity (Stuber *et al.*, 2013; Knotts and Griffen, 2016; Maskrey *et al.*, 2018).

By systematically analysing these differences, we can better understand how giant pandas cope with environmental challenges through behavioural and physiological mechanisms. These insights support targeted interventions to improve population-level adaptability and fitness and build upon a substantial body of research on animal personality by advancing a broader behavioural syndrome framework specific to giant pandas. In turn, they could inform conservation and restoration programs and underscore the necessity of recognizing adaptive behavioural diversity in wildlife to confront the global biodiversity crisis.

## Materials and Methods

### Ethics statement and study subjects

All procedures were non-invasive and approved by the institutional ethics board (Permit: NO. SCU240908001). The captive cohort comprised 12 giant pandas (9 males, 3 females; 2–16 years old; Table 1) housed at the China Conservation and Research Centre for the Giant Panda (CCRCGP), Dujiangyan Base. The sample was male-biased because several adult females were unavailable for testing due to concurrent nursing/reproductive management. Pandas were housed in enclosures with connected inner (two 3 × 3 m rooms used for isolated testing) and outer vegetated areas (Fig. S1). All 12 individuals completed the Resource Depletion and String-Pulling tasks, while one individual was excluded from the MAB analyses (*N* = 11) due to an unscheduled husbandry relocation.

**Table 1:** Characteristics and behavioural metrics of the 12 captive giant pandas

**Panda name**	**Reactive**	**Learning rate**	**Learning impediment**	**Exploratory adaptiveness**	**Handedness**	**DRS cross**	**DRS parallel**	**fGCM(ng/g)**	**Age (years)**	**Gender**	**Weight (kg)**
E Mei	3.12	0.59	−0.25	−1.71	L	105	1	442.5	3	F	101
Lin Xi	−0.25	−0.66	−0.13	−1.23	L	86	119	474.08	2	M	69
Mao Mao	−0.78	0.13	0.8	−0.54	L	17	1	302.48	2	M	60
Mu Ye	1.71	0.46	0.77	−1.17	L	151	79	489.22	4	M	108
Nong Nong	1.87	−1.04	2.47	−1.46	N	83	98	410.24	3	M	112
Qing Qing	0.4	1	−1.73	0.8	L	16	17	326.42	8	M	120
Su Yang	−1.91	−0.53	1.65	1.54	R	32	35	165.94	4	M	100
Xing Ye	−1.8	1.09	−0.4	1.77	R	48	111	256.52	4	M	119
Xing Yue	−0.33	0.79	−1.9	−0.08	L	137	1	321.96	4	M	121
Yun Yun	−0.47	−2.35	2.38	1.51	L	23	7	166.84	14	M	135
Zhu Ling	−1.57	0.53	−2	1.85	R	45	2	187.48	16	F	116
Ling Lang			−1.66	−1.28	R	27	19	479.33	5	F	99

a ^a^DRS: higher values indicate poorer performance (i.e. more trials required to reach criterion)

a ^a^fGCM: (ng/g), quantified from the standardized five-day sampling window

a ^a^MAB indices: reactive score, learning rate

a ^a^Resource depletion indices: learning impediment, exploratory adaptiveness

a ^a^String-pulling indices: DRS, handedness (L/R/N; derived from rope-pulling forelimb use)

a ^a^Empty cells indicate that the corresponding metric was not available for that individual

### Study design and timeline

The captive component (July 2023 to February 2024) began with a 5-day baseline fGCM sampling window (26–30 July 2023) conducted early morning (09:00–11:00) to minimize diurnal variation. Three behavioural paradigms were then conducted sequentially: Resource Depletion Test (July–September 2023); String-Pulling Task (October–December 2023) and MAB Test (January–February 2024).

To minimize seasonal or motivational confounds, all trials were embedded within the daily feeding routine (09:00, 11:00, 13:30, 16:00) using highly preferred, non-seasonal supplemental treats (e.g. carrots). Subjects were never food deprived. A rotational schedule allowed three to four testing days per week per subject (Fig. S2). Each paradigm was completed within a relatively restricted block (2–3 months), and our primary behavioural metrics were derived within-task (trial-structured) and then summarized at the individual level, reducing sensitivity to slow seasonal drift. All behavioural sessions were video-recorded for frame-by-frame scoring.

The field component was conducted from April to May 2023 in the Qianfoshan National Nature Reserve, Sichuan, China (103°56′E to 104°18′E, 31°37′N to 31°48′N, Fig. 2), where faecal samples were collected for individual identification and fGCM analyses.

### Faecal sampling and glucocorticoid metabolite assays (captive and wild)

To assess stress-related endocrine output, we quantified fGCM concentrations. For captive pandas, fresh faecal samples were immediately vacuum-sealed and stored at −20°C. For wild samples, endocrine subsamples were sealed and frozen at −20°C on the day of collection (see Field sampling). Hormones were extracted from lyophilized, pulverized, and sifted faeces using 90% ethanol following established protocols (Wasser *et al.*, 1994). fGCM concentrations were quantified via enzyme immunoassay (EIA) using a cortisol antibody (R4866; C. Munro, UC Davis) previously validated for giant pandas (Kersey *et al.*, 2010). Assay performance was monitored using high and low in-house controls on each plate; intra- and inter-assay CVs were 4.99% and 6.05% (see Table S1). Given the short, standardized five-day sampling window, we treat these fGCM values as study-entry baseline proxy concentrations (hereafter, ‘baseline’).

### Behavioural assays (captive)

We employed three experimental paradigms to assess personality- and cognition-related traits.

### MAB test

**Figure 1:**
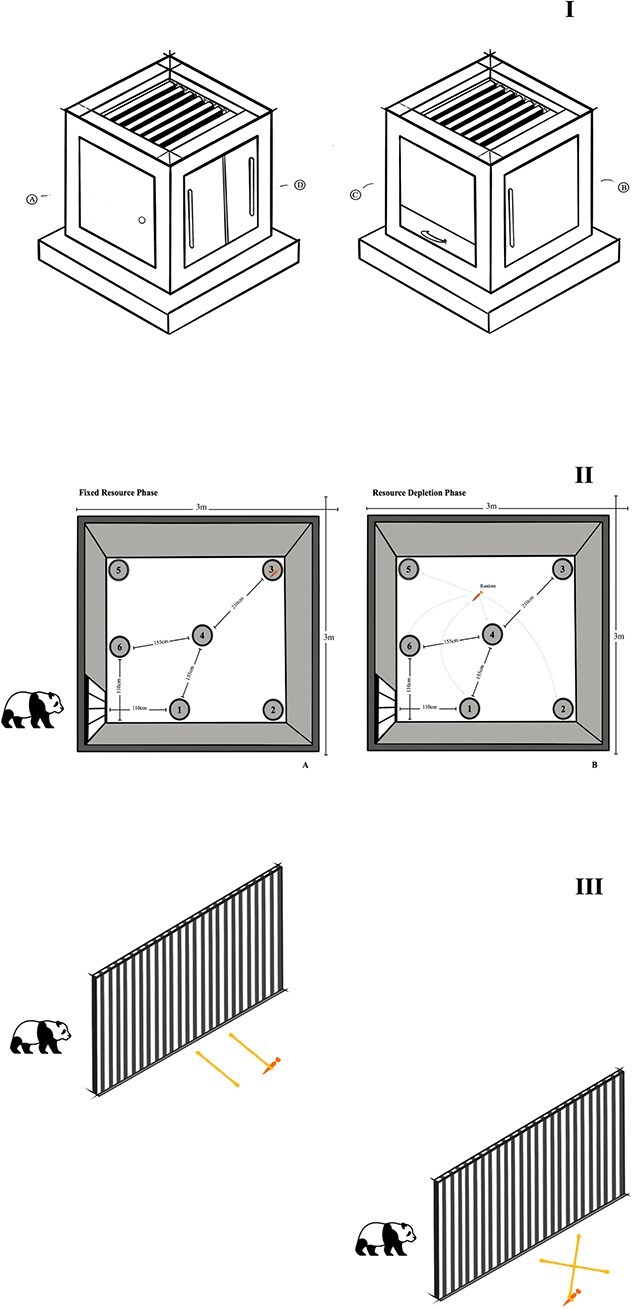
Experimental setups for behavioural tests 1–3 on Giant Pandas. Panel I: The MABs utilized in the experiments. (A) Push flap solution, (B) pull flap solution, (C) sliding doors solution and (D) drawer solution. Panel II: Setup of the resource depletion test. A depicts the experimental arrangement during the fixed resource phasE, where food is consistently placed under bowl no. 3. B shows the setup for the resource depletion phase, illustrating the altered conditions where food is randomly placed under three of the other five bowls, with the previously fixed location (NO.3) no longer containing food. Panel III: Setup of the String-Pulling Experiment. Illustrates the experimental arrangements for both phases of the string-pulling test: phase parallel (first stage) and phase cross (second stage)

To quantify problem solving together with personality-linked responses to novelty and constraint (e.g. neophobia, persistence, and inhibitory control), we used a four-solution MAB with distinct opening mechanisms (push, pull, slide, and drawer; apparatus details in Fig. 1 and Appendix SI). Testing consisted of a familiarization stage (all solutions available; two to five sessions) followed by four phases in which solutions were sequentially blocked. A solution was considered learned after three successful trials; if no solution was opened in three consecutive trials, the phase ended.

We quantified trial-level measures of (i) persistence (proportion of trial time spent actively interacting with the apparatus), (ii) inhibitory control (inverse; time allocated to previously learned but blocked solutions relative to total exploration time), and (iii) efficiency/latency to success (recorded but excluded from downstream analyses due to negligible repeatability; see Statistical Analysis). To characterize learning dynamics, we also extracted exploration duration per trial and modelled its change across trials; individual learning rate was calculated as the average within-phase slope of exploration duration against trial number, standardized across phases. In addition, we extracted one-time, first-encounter measures of neophobia (latency from trial start to first contact with the MAB during the first familiarization trial) and exploratory diversity (behavioural repertoire expressed toward the MAB on first exposure), as well as task completion (total number of trials completed). Full scoring definitions and ethogram are provided in Table S2 and Appendix SI.

### Resource depletion test

Adapted from the radial arm maze (Olton and Samuelson, 1976; Olton and Paras, 1979; Tarou *et al.*, 2004; Perdue *et al.*, 2011), this test quantified spatial learning and flexibility (Fig. 1). Six overturned paper bowls were arranged in a fixed spatial configuration that remained constant across trials; only reward placement varied between phases.

The task comprised two sequential phases. In the fixed resource phase, food was consistently placed under a single fixed bowl (the learned location; Bowl No. 3) for a minimum of 12 trials and up to 20 trials. Individuals were considered to have learned the spatial rule when they located the reward with ≤2 picks in three consecutive trials; subjects failing to meet this criterion within 20 trials were classified as not having learned the task. In the subsequent Resource Depletion Phase, the previously rewarded bowl was no longer baited, and food was randomly distributed under three of the remaining five bowls across 15 trials, requiring subjects to inhibit perseverative returns to the learned location and update their search strategy.

We extracted two classes of metrics (Table S3 for full details). Learning performance was indexed by pick count (number of bowl inspections/picks required to locate reward within a trial), trials to learn (trial number at which the learning criterion was first met) and delayed fixation trials (DFT, trials required to stop returning to the previously rewarded location). Flexibility and memory-related behaviours were quantified using working memory errors (WME) (within-trial revisits to previously inspected locations), learned-location visit ratio (LLVR = visits to learned location/total visits), learned-location stay duration (LLSD = time at learned location/total exploration time), exploration accuracy (accuracy = 1 − [unsuccessful picks/total picks]) and total locations visited (TLV) as an index of search breadth. Detailed procedures and scoring rules are provided in Appendix SI.

### String-pulling task

To evaluate physical cognition and lateralization, naive pandas were tested with a two-string choice paradigm in which only one rope was baited (Hofmann *et al.*, 2016; Wang *et al.*, 2019). Two phases were administered: a parallel configuration (two 1 m hemp ropes laid in parallel) followed by a crossed configuration (two 1.2-m ropes arranged in a crossed pattern; Fig. 1). In each trial, a keeper distracted the subject at the far end of the enclosure during setup to prevent the animal from observing baiting, after which the subject was released and allowed to choose. If the subject selected the unbaited rope, no reward was provided, and the apparatus was reset for the next trial.

Each configuration phase comprised up to 150 trials. Learning was defined as ≥80% success in three consecutive 10-trial moving windows; trials to criterion were recorded as deductive reasoning score (DRS), with non-learners assigned DRS = 151.

### Field study, faecal sampling (wild) and genotyping

**Figure 2:**
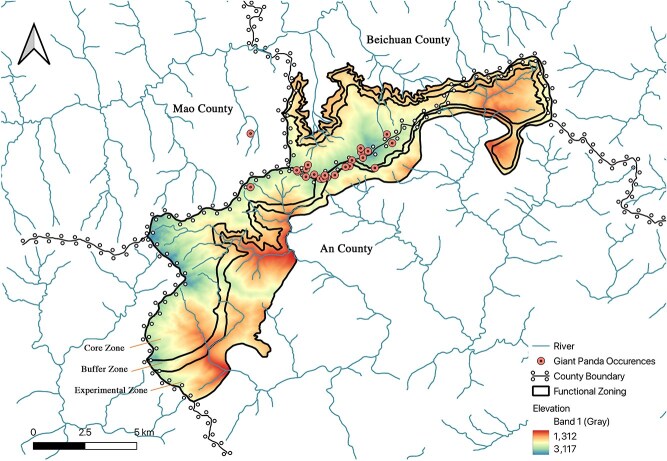
Plot of field faecal sample collection

Fieldwork was conducted from April to May 2023 in the Qianfoshan National Nature Reserve, Sichuan, China (Fig. 2). Ranger-guided transects were used to locate relatively fresh giant panda faeces; freshness was assessed using a published rubric (Deng *et al.* (2014); see Appendix SI), and only samples estimated to be <7 days old were retained for fGCM analyses (Millspaugh and Washburn, 2004; Kai *et al.*, 2014). To minimize human DNA contamination, samples were collected using disposable gloves (changed between samples) and immediately split into paired subsamples: the external mucous layer was preserved in 99% ethanol for genotyping, whereas the inner faecal core was sealed (without ethanol) and frozen at −20°C for endocrine analysis. In total, 42 faecal piles were collected. Genomic DNA was extracted from the ethanol-preserved subsamples using the TIANamp Stool DNA Kit (Tiangen Biotech, Beijing); samples with low-quality DNA (assessed by agarose gel electrophoresis) were excluded prior to PCR amplification.

Individual identification was performed using eight tetranucleotide microsatellite loci (Table S4) with standard multi-tube genotyping and quality control (see Appendix SI). Marker discriminatory power was evaluated using PID and PID(sib), targeting PID(sib) < 0.01(Waits *et al.*, 2001). This workflow yielded nine wild individuals. Behavioural experiments were conducted only on captive individuals; wild data were used for endocrine–disturbance association analyses.

Human disturbance was quantified as the shortest surface distance (SSD) from each sample location to the nearest road or agricultural land. Disturbance layers were derived from a 30-m land-use layer Yang and Huang (2023) and OpenStreetMap road data (OpenStreetMap Contributors, 2024), while topographical data were obtained from the ALOS World 3D-30 m (AW3D30) DEM (Japan Aerospace Exploration Agency, 2021). To ensure reproducibility and account for terrain relief, disturbance layers were converted into 30-m point matrices, and SSD was computed using the ‘Profiles from lines’ tool in QGIS v3.36.3. This method calculated the actual ground distance along the terrain profile rather than the Euclidean distance, providing a more ecologically valid metric of physical accessibility.

### Statistical analyses

For repeated behavioural measures (e.g. MAB persistence and inhibitory control; resource-depletion flexibility metrics), we fitted Gaussian linear mixed models (LMMs) with individual identity as a random intercept to quantify among-individual variance while accounting for trial structure. Fixed effects included trial number (and phase where applicable), sex, age class (subadult vs adult), and body mass; predictors were retained as covariates where they improved model fit or were associated with the response. Repeatability was quantified as intraclass correlation coefficients (ICC) from the corresponding random-intercept models, with significance determined via Likelihood Ratio Tests (LRT) comparing models with and without the random effect. Uncertainty was estimated via bootstrap confidence intervals. Following established guidelines (Koo and Li, 2016), only metrics demonstrating significant repeatability (*P* < 0.05) were averaged to extract individual mean scores for downstream analyses.

To summarize correlated behavioural variables, we examined pairwise Spearman correlations and applied principal component analysis (PCA) to standardized variables when strong collinearity was present (|ρ| > 0.7). Specifically, we derived composite behavioural scores for both experimental paradigms to capture underlying personality and cognitive dimensions. For the MAB test, we extracted a primary component labelled ‘reactive score’ from correlated metrics (neophobia, persistence, inhibitory control, and efficiency); higher scores on this axis represented a behavioural syndrome characterized by high neophobia, low persistence, and reduced inhibitory control. For the Resource Depletion Test, we derived two components based on theoretically clustered metrics (detailed in Appendix SI): ‘Learning Impediment’ (derived from Pick Count, Trials to Learn, and DFTs), where higher scores reflected slower rule acquisition; and ‘Exploratory Adaptiveness’ (derived from LLSD and Exploration Accuracy), describing the trade-off between thoroughness and flexibility. Variables showing weak collinearity (e.g. WME, TLV, LLVR) were analysed as independent predictors. Adequacy checks (KMO/Bartlett) and component retention criteria are detailed in Appendix SI.

Associations between baseline fGCM concentrations and behavioural traits (raw or PCA scores) were tested using linear models (GLMs with identity link), with sex, age class, and body mass included as covariates where relevant.

For wild samples, we assessed the relationship between fGCM and human disturbance (SSD) using (i) an individual-mean model relating each identified individual's mean fGCM to mean SSD, and (ii) within-individual models for individuals with ≥3 repeated samples, relating sample-level fGCM to SSD to capture short-term covariation. Full model specifications, diagnostics, and supplementary analyses are provided in Appendix SI.

All analyses were performed using R 4.2.2 (Wickham, 2009; Bates *et al.*, 2015; Lüdecke and Lüdecke, 2019; Wickham *et al.*, 2019; R Core Team, 2022) and Python 3.10.9 (Python Core Team, 2024).

## Results

### Faecal hormone analysis

We quantified fGCM concentrations in 60 faecal samples (five consecutive samples per individual) from the 12 captive subjects. Individual means ranged from 165.94 to 489.22 ng/g (Table 1; raw values in Table S5). These averaged values were utilized as the study-entry baseline profiles for subsequent behavioural analyses.

### MAB test performance and learning outcomes

Eleven pandas completed the MAB test, successfully learning all four solutions. To quantify learning progress, we modelled exploration duration as a function of trial number using LMM. Exploration duration decreased significantly across trials (*P* < 0.001), indicating increasing proficiency. The derived individual learning-rate index (Table 1) was not significantly associated with baseline fGCM concentrations (β = 0.0018, *P* = 0.521).

### Personality traits

Persistence and inhibitory control demonstrated significant repeatability (χ^2^ = 16.996, *P* < 0.001, ICC = 0.318, 95% CI [0.031, 0.621], and χ^2^ = 5.584, *P* < 0.05, ICC = 0.416, 95% CI [0.031, 0.629], respectively), whereas efficiency lacked repeatability (ICC = 0) and was excluded from further analyses.

Persistence, neophobia, and inhibitory control were highly correlated (|ρ| > 0.7; Table 2), and they were subjected to PCA (KMO = 0.61; Bartlett's *P* = 0.001). A single principal component (PC1) was retained according to the Kaiser criterion (Greggor *et al.*, 2015) and the Scree test (Bates *et al.*, 2015), explaining 81.0% of the total variance (Fig. S3). We labelled this PC1 as the ‘reactive’ axis. It was driven by positive loadings for neophobia (0.56) and inversed inhibitory control (0.62), and a negative loading for persistence (−0.55; Fig. S4). Higher scores indicate a more reactive coping style along the proactive–reactive axis (Koolhaas *et al.*, 1999; Sih *et al.* 2004), characterized by high neophobia, low inhibitory control, and reduced persistence.

**Table 2:** Correlation matrix among subjects’ MAB scores

	Exploratory diversity	Neophobia	Persistence	Inhibitory
Exploratory diversity	1	−0.36	0.40	−0.35
Neophobia		1	−0.47	0.77
Persistence			1	−0.77
Inhibitory				1

**Figure 3:**
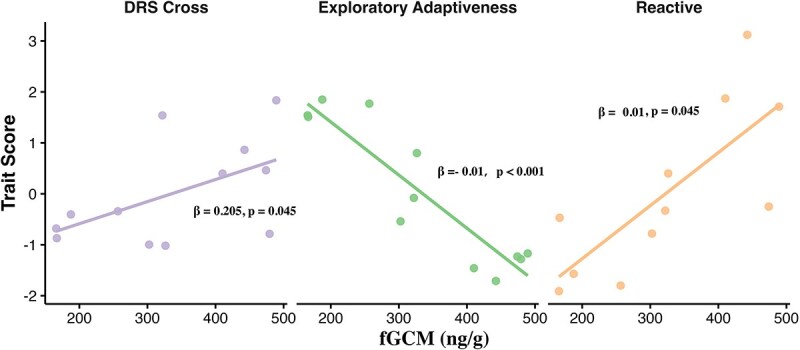
Relationships between baseline fGCM concentrations (ng/g) and trait scores derived from PCAs, as well as the standardized DRS in the cross phase. All traits shown exhibited significant associations with fGCM concentrations in captive giant pandas. Higher DRS scores indicate lower causal inference ability

The reactive score was significantly positively correlated with total experiments completed (ρ = 0.825, *P* = 0.002) but not with the learning rate (*P* = 0.853). Individual background factors (sex, age class and body weight) did not significantly affect reactive scores or exploratory diversity (all p > 0.05; detailed statistics in Table S8). Crucially, GLMs revealed that baseline fGCM concentrations significantly positively predicted reactive scores (β = 0.01, *P* < 0.001; Fig. 3) and negatively predicted total experiments completed (β = −0.006, *P* < 0.001). However, baseline fGCM did not predict exploratory diversity (*P* = 0.561).

### Resource depletion test

All 12 giant pandas (4 females, 8 males; Table 1) successfully completed the Resource Depletion Test. All repeated metrics—Pick Count, LLVR, LLSD, Exploration Accuracy, WME and TLV—exhibited significant intra-individual repeatability (ICC range, 0.081–0.300; all *P* < 0.05; see Table S7). Because WME, TLV and LLVR lacked strong correlations with other metrics (|ρ| < 0.7; Tables 3 and 4), they were analysed independently and excluded from subsequent PCAs.

**Table 3:** Correlation matrix of subjects’ learning scores

	Pick count	Trials to learn	DFT	WME
Pick count	1	0.77	0.59	0.69
Trials to learn		1	0.80	0.56
DFT			1	0.33
WME				1

a ^a^DFT: the number of trials required for a subject to not return to the initially learned feeding point until at least three picks in three consecutive trials.

a ^a^WME: revisits to any location within the same trial, identifying repeated explorations of the same point.

**Table 4:** Correlation matrix among subjects’ personality scores

	LLVR	LLSD	Exploration accuracy	TLV
LLVR	1	−0.09	−0.20	−0.44
LLSD		1	−0.81	0.34
Exploration accuracy			1	−0.17
TLV				1

a ^a^LLVR: frequency of visits to the learned location relative to total visits

a LLSD: proportion of time spent at the learned location against the overall exploration time.

a ^a^TLV: total number of locations visited during the trials.

We conducted separate PCAs for the remaining learning and personality metrics (both KMO ≥ 0.50, Bartlett’s *P* ≤ 0.001). For learning metrics (Pick Count, Trials to Learn, DFT), the first principal component explained 81.4% of the variance (Fig. S7). Driven by positive loadings across all three metrics (≥0.56; Fig. S8), higher values reflect slower rule acquisition, prompting us to label this axis ‘Learning Impediment’. For personality metrics (LLSD, Exploration Accuracy), a single component explained 90.7% of the variance (Fig. S5). Driven by a negative loading for LLSD (−0.71) and positive for exploration accuracy (0.71) (Fig. S6), higher scores indicate greater flexibility and focused search patterns, termed ‘Exploratory adaptiveness’.

Individual background factors did not significantly influence any metrics from this test (all *P* > 0.07; detailed statistics in Table S8). Crucially, GLMs revealed that constitutive fGCM concentrations significantly negatively predicted Exploratory Adaptiveness (β = −0.01, *P* < 0.001; Fig. 3), but did not predict other metrics.

### String-pulling tasks

All 12 individuals met the learning criteria in the parallel setting, and 11 succeeded in the cross setting. The number of trials required to reach the criterion did not differ significantly between the two setups (Wilcoxon signed-rank test: *W* = 23, *P* = 0.233).

Individual background factors did not significantly affect DRS in either phase (all *P* > 0.05; detailed statistics in Table S8). GLMs showed that baseline fGCM concentrations significantly predicted DRS only in the cross phase (β = 0.205, *P* = 0.045; Fig. 3), where lower fGCM correlated with faster acquisition (lower DRS). Regarding laterality, 11 of 12 subjects exhibited significant and consistent handedness across both phases, with only one subject lacking a significant preference (*Z* = 1.03; Table 1).

### Cross-experiment analysis of behavioural metrics

Reactive scores significantly negatively predicted exploratory adaptiveness (β = −0.703, *P* < 0.001). Learning rate (from the MAB test) significantly negatively predicted learning impediment score (β = −1.216, *P* < 0.001). However, neither learning impediment score nor learning rate significantly predicted DRS in either string-pulling phase (all *P* > 0.05).

Learning Impediment Score was significantly positively associated with both WME (β = 0.887, *P* = 0.024) and TLV (β = 0.592, *P* = 0.011).

Finally, the absolute Handedness Index (|HI|) positively predicted the reactive score (*N* = 10, β = 26.89, *P* = 0.028) and negatively predicted exploratory adaptiveness (*N* = 11, β = −23.74, *P* = 0.026). Furthermore, right-handed individuals scored significantly lower on the reactive axis than left-handed individuals (*t* = 2.96, *P* = 0.018).

### Field study

#### Individual identification

Of the 42 collected faecal samples, 34 yielded sufficient DNA, and 27 were confirmed via mitochondrial D-loop amplification. Following microsatellite genotyping and quality control, 18 samples were successfully genotyped. Across these 18 samples, we detected 43 alleles across eight loci (mean = 5.4 alleles per locus; range = 2 to 9; Table S6). The five loci with the highest polymorphic information content (PIC; Table 5) provided sufficient discriminatory power (sibling probability, PID(sib) = 0.0072), successfully identifying nine distinct wild giant pandas.

**Table 5:** Genetic diversity parameters of microsatellite loci in wild Giant Pandas

Locus	Private alleles	Alleles	Ho	He	PIC
gpz20	9	9	0.214	0.897	0.85
gpz6	6	8	0.308	0.883	0.831
gpz47	5	7	0.944	0.814	0.764
GPL8	4	4	0.125	0.742	0.645
GPL60	6	6	0.063	0.736	0.668
gpy5	4	4	0	0.762	0.678
GPL29	3	3	0	0.594	0.477
gpy20	2	2	0	0.173	0.152

### fGCM concentrations and individuality in wild giant pandas under field conditions

We calculated the SSD for each faecal sample (a total of 18 samples) from the identified individuals, representing the shortest distance to human disturbance sources while accounting for elevation factors (Table 6). Among the nine distinct giant panda individuals identified, we further analysed the relationship between SSD and fGCM concentrations at both intra-individual and inter-individual levels. For the intra-individual analysis, we examined the SSD and fGCM concentrations of individuals with three or more repeated faecal samples. For the inter-individual analysis, we assessed the relationship between SSD and the average fGCM concentrations across all identified individuals.

**Table 6:** fGCM concentrations, habitat elevation, and SSD of 18 faecal samples collected from 9 wild Giant Pandas

Sample ID	Elevation (m)	fGCM (ng/g)	Wild individual ID	SSD (km)
Q-D-9	2708	9.3	G	7.05
Q-D-10	2733	16.6	A	27.94
Q-D-11	2712	119.8	A	7.05
Q-D-12	2695	93.1	A	9.85
Q-D-13	2713	8.1	H	22.87
Q-D-14	2698	40.6	B	8.90
Q-D-15	2713	27.5	I	19.13
Q-D-16	2675	173.9	C	28.95
Q-D-17	2695	120.2	B	10.94
Q-D-18	2676	119.8	H	5.07
Q-D-20	2706	8.5	E	6.05
Q-D-21	2703	43.6	D	27.94
Q-D-25	2652	31.2	D	8.90
Q-D-26	2594	29.4	A	19.13
Q-D-27	2593	21.7	A	27.60
Q-D-29	2663	83.1	A	8.84
Q-D-30	2523	12.1	A	32.71
Q-D-32	2609	103.9	F	25.88

Note: Samples are grouped by Wild individual ID.

**Figure 4:**
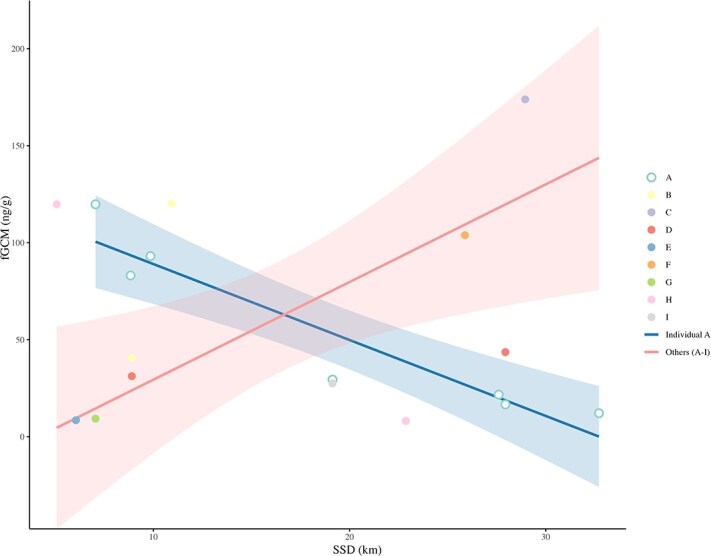
Relationship between fGCM concentrations and SSD to human disturbance in wild giant pandas. The blue line represents the significant negative intra-individual trend for the single repeatedly sampled panda (Individual A). The red line indicates the significant positive inter-individual trend across the sampled population (Others A–I). Shaded areas represent 95% confidence intervals

Specifically, in the intra-individual fGCM concentrations analysis, only one wild giant panda met the criterion of three or more repeated samples. A linear model for this individual revealed a significant negative correlation between SSD and fGCM concentrations (*N* = 7, β = −3.92, *P* = 0.001, Fig. 4). For inter-individual analysis, we calculated the average fGCM concentrations for each of the nine individuals. Linear regression results showed that as SSD increased, the average fGCM concentrations of individuals also significantly increased (*N* = 9, β = 5.035, *P* = 0.017, Fig. 4).

## Discussion

**Figure 5:**
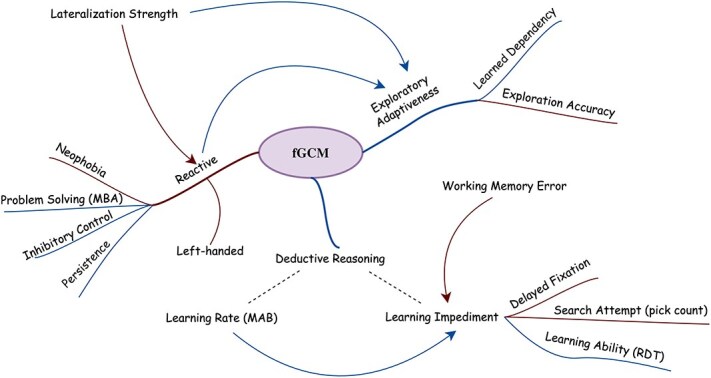
The network plot represents the interconnections among behavioural, cognitive, and physiological traits in captive giant pandas. Blue edges denote significant negative associations, red edges indicate significant positive associations, and dashed edges represent non-significant relationships. ‘MAB’ refers to the MAB experiment, while ‘RDT’ denotes the resource depletion test

Our research integrates three behavioural experiments with a field study, providing initial evidence that constitutive fGCM concentrations covary with specific personality dimensions and cognitive performance in giant pandas. We further identify significant associations between behavioural lateralization and panda personalities. Proactive individuals, characterized by lower constitutive fGCM concentrations, show greater exploratory behaviour and a coping-related behavioural profile that may be advantageous when environmental conditions fluctuate. Together, these results offer an integrative framework for behavioural syndromes in this species, linking patterns observed under captive experimental settings (Fig. 5) with field-relevant variation among wild individuals. Such mechanistic insight helps anticipate which behavioural phenotypes may be more resilient, or more vulnerable, during reintroduction and rapid habitat change, thereby informing individual-based conservation and management strategies.

### Captive giant panda behavioural experiments

The MAB paradigm effectively distinguished between individuals along the proactive-reactive axis. Specifically, more reactive individuals exhibited higher baseline fGCM concentrations, increased neophobia, reduced inhibitory control, less persistence, and lower task completion capabilities. This aligns with findings in other species where more reactive individuals tend to be more sensitive to stressors, leading to more cautious and less exploratory behaviour (Sih *et al.* 2004; Mazza *et al.*, 2019). While our correlational data cannot establish causality, the fGCM–personality link is likely bidirectional: inherent physiological differences in HPA-axis regulation may predispose individuals to specific behavioural phenotypes, while the stress associated with reactive behavioural strategies could conversely elevate fGCM concentrations. Clarifying this feedback loop will be essential in future studies.

Regardless of the precise primary driver, the MAB results demonstrate that giant pandas have remarkable problem-solving skills and learning flexibility. Specifically, all 11 subjects successfully mastered all four distinct opening mechanisms, and exploration duration decreased significantly across trials, indicating increasing task proficiency with experience. Notably, performance in our cohort is at least as strong as—and may exceed—that reported for other large-brained mammals tested with the same general MAB framework: for example, spotted hyenas (*Crocuta crocuta*; Johnson-Ulrich *et al.* (2018). Although direct cross-study comparisons should be made cautiously because apparatus details, training criteria, and trial structure can differ, the uniformly high multi-solution mastery observed here supports the conclusion that giant pandas can rapidly learn and flexibly update action–outcome strategies in a multi-solution problem context.

The Resource Depletion Test revealed a negative correlation between fGCM concentrations and Exploratory Adaptiveness. Contrary to past studies across various vertebrate taxa, which posited that reactive individuals with higher glucocorticoid levels should be slower, more thorough, and more flexible in exploring new situations (Sih *et al.* 2004; Geffroy *et al.*, 2020; Cockrem, 2022), our study shows that proactive pandas with lower fGCM concentrations demonstrated exceptional adaptability to changing food locations. This is further supported by the negative correlation between the MAB reactive score and exploratory adaptiveness. We suggest that stable food supplies in captivity may foster proactive phenotypes that excel in structured variability. By recovering more rapidly from initial stressors (Carere and Van Oers, 2004; Ruiz-Gomez *et al.*, 2011), proactive pandas can quickly exploit new resources without the acute stress responses that often trigger cognitive dissonance in highly reactive individuals (Sandi, 2013). Thus, while typically seen as a disadvantage in unpredictable wild environments, proactive strategies become highly advantageous in controlled, predictable settings (Altschul *et al.*, 2017; Mazza *et al.*, 2018; Rasolofoniaina *et al.*, 2021).

Crucially, the interpretation of lower fGCM concentrations demands nuance within the broader framework of conservation physiology. Although lower glucocorticoid output is sometimes taken to indicate ‘low stress’ or ‘good welfare’ (Millspaugh and Washburn, 2004; Palme, 2019), similar baseline values can arise from different regulatory regimes across contexts and timescales. Specifically, under chronic or repeated challenges, reduced glucocorticoid output may also be consistent with HPA-axis dysregulation or ‘blunting’ (Busch and Hayward, 2009; Dickens and Romero, 2013). Therefore, the strongest inference from our data is relational rather than normative, implying that endocrine profiles should be interpreted alongside functional outcomes. In our study, the low-fGCM (proactive) individuals were characterized not by behavioural withdrawal, but by sustained exploratory engagement and effective problem-solving. This pattern is more compatible with efficient baseline regulation and recovery in the present setting (Korte *et al.*, 2005) than with an overt failure to respond. Nevertheless, distinguishing resilience from potential hypo-responsiveness will require higher-frequency longitudinal sampling, concurrent measures of acute reactivity/recovery, and additional biomarkers (e.g. immune or oxidative markers) linked to fitness or welfare outcomes.

In the string-pulling task, 11 of 12 pandas mastered both the parallel and the more challenging crossed configurations, demonstrating a clear comprehension of causal action–reward connections (Video S1; Hofmann *et al.* (2016). Such performance surpasses many species, including azure-winged magpies (*Cyanopica cyanus*) (Wang *et al.*, 2019), Western scrub-jays *(Aphelocoma californica)* (Hofmann *et al.*, 2016), as well as domestic cats *(Felis catus)* (Whitt *et al.*, 2009) and dogs *(Canis lupus familiaris)* (Osthaus *et al.*, 2005). Furthermore, the lack of significant performance differences between the parallel and crossed phases indicates that individual deductive reasoning abilities in giant pandas remain remarkably robust and consistent across varying levels of task complexity (Heinrich and Bugnyar, 2005).

Interestingly, while baseline fGCM did not directly predict performance in the MAB or Resource Depletion Test, individuals with lower fGCM performed significantly better in the more cognitively demanding cross-setting of the string-pulling task. This disparity likely reflects the differing cognitive and stress loads of these paradigms. The MAB and resource depletion tasks primarily assess memory and flexibility through relatively straightforward cognitive processing. In contrast, the crossed string-pulling task requires complex spatial and logical reasoning, imposing a higher challenge load and a greater risk of repeated failure, which more strongly engages the stress-response system. Under such acute pressure, higher stress can render individuals insensitive to outcome values and resistant to updating action-outcome contingencies (Dias-Ferreira *et al.*, 2009; Sandi, 2013). Therefore, pandas with lower baseline fGCM, indicative of a proactive coping style and efficient HPA-axis regulation, are better equipped to preserve cognitive control and make effective decisions. Ultimately, baseline fGCM serves as a physiological signature of coping style that becomes most predictive when tasks are sufficiently challenging. Future studies jointly quantifying baseline output, acute reactivity and recovery trajectories will further clarify how glucocorticoid profiles map onto cognitive performance across contexts.

The negative relationship between MAB learning rate and the resource depletion learning impediment score underscores a robust, cross-context consistency in individual learning capabilities. Furthermore, higher WME and TLV correlated with greater learning impediment. This suggests that pandas adopting a broader, trial-intensive exploratory strategy may sacrifice learning efficiency. These results support the hypothesis that animals with ‘fast’ behavioural types may exhibit cognitive styles emphasizing speed over accuracy (Sih and Del Giudice, 2012). While evidence for cognition–personality trade-offs is accumulating across species (Exnerová *et al.*, 2009; Amy *et al.*, 2012; Brust *et al.*, 2013; Guenther *et al.*, 2014), our findings provide crucial empirical support for the cross-contextual stability of these integrated traits (Guillette *et al.*, 2015).

Our lateralization analysis revealed that 11 of 12 pandas displayed a clear hand preference, with left-handed individuals scoring significantly higher on the Reactive axis. This aligns with the valence hypothesis of brain lateralization, which posits that the right hemisphere (controlling the left side of the body) is specialized for processing negative emotions and withdrawal behaviours (Rogers and Andrew, 2002; Demaree *et al.*, 2005; Rogers, 2015; Rogers, 2017). Accordingly, left-handed vertebrates are typically more prone to fear and anxiety (Westergaard *et al.*, 2003; Gordon and Rogers, 2010). Beyond directional preference, the absolute strength of lateralization was positively associated with reactivity and negatively with exploratory adaptiveness, mirroring patterns in other mammals (Marshall-Pescini *et al.*, 2013). This suggests a conserved neurobiological link between strong motor lateralization and behavioural rigidity. For a large, solitary forager whose ecological strategy prioritises energy conservation and risk avoidance, such rigidity could reduce cognitive flexibility in responding to novel environmental changes or resource competition. Ultimately, these results provide valuable mechanistic insight into the interplay between lateralization, personality and adaptive cognitive flexibility in this iconic species.

### Giant pandas under field conditions

At the inter-individual level, pandas with higher average fGCM concentrations were located farther from human infrastructure. This aligns with our captive findings: reactive phenotypes (higher baseline fGCM) exhibit greater risk sensitivity and spatial avoidance of disturbance, whereas proactive individuals (lower fGCM) may be more disturbance-tolerant (Naguib *et al.*, 2013). Conversely, our intra-individual data, supported by previous findings (Deng *et al.*, 2014), revealed that when a specific individual moves closer to human activity, its fGCM elevates relative to its own baseline. This divergence suggests a critical ecological trade-off: while proactive phenotypes are more likely to exploit human-modified habitats, proximity to anthropogenic activity still elicits a physiological stress response. Although larger datasets incorporating movement tracking are needed, these findings highlight the necessity of integrating behavioural syndromes into conservation planning amidst rapid habitat modification (Lenoir and Svenning, 2014).

The substantially lower absolute fGCM concentrations in wild samples compared to captive baselines must be interpreted cautiously and do not inherently imply lower stress in the wild (Kai *et al.*, 2014). This discrepancy likely arises from several unavoidable methodological and ecological confounds. First, despite our <7-day freshness threshold, environmental exposure (temperature, precipitation, microbial metabolism) inevitably degrades field-collected faecal metabolites (Millspaugh and Washburn, 2004; Goymann, 2012). Second, marked differences between captive and wild diets significantly alter gut passage rates, faecal water content and matrix composition, directly influencing extraction efficiency (Goymann, 2012). Finally, unmatched seasonality (April–May vs. July) and unknown demographic structures in the wild cohort may further confound absolute comparisons (Romero, 2002; Kersey *et al.*, 2010; Goymann, 2012). Consequently, our robust inferences rely strictly on within-context relationships (i.e. fGCM–behaviour links in captivity; fGCM–disturbance covariation in the wild) rather than cross-setting absolute baseline comparisons.

Our findings reveal that proactive pandas—characterized by bold, exploratory behaviour and lower baseline fGCM—exhibit greater stress resilience. Although we did not directly measure dispersal or translocation outcomes, these personality–stress linkages provide a mechanistic foundation for understanding spatial ecology. In other taxa, traits such as exploration and boldness strongly predict dispersal ability and habitat use (Dingemanse *et al.*, 2003; Cote *et al.*, 2010; Berner and Thibert-Plante, 2015; Knotts and Griffen, 2016). Yet, personality is rarely incorporated into panda spatial studies, reflecting a broader taxonomic bias in conservation behaviour research (Collins *et al.*, 2023). Neglecting these individual variations may obscure critical patterns of habitat exploitation and hamper effective management.

Integrating behavioural syndromes is particularly pertinent for active conservation interventions, such as China's giant panda reintroduction programmes (Wei *et al.*, 2015a, 2015b; Huang, 2016; Yang *et al.*, 2018; Wang, 2019). Selecting release candidates based on behavioural plasticity and stress resilience, alongside traditional genetic representation, could significantly improve initial establishment and long-term population viability (Fogarty *et al.*, 2011; Mackinlay and Shaw, 2023; Wu *et al.*, 2024). Crucially, this principle also extends to the management of the global ex situ population, including international loans and transfers under cooperative breeding and conservation agreements (often referred to as ‘panda diplomacy’ in public discourse). This is especially relevant for giant pandas, a niche-specialist flagship species that is routinely transported across large climatic and environmental gradients. Relative to generalist taxa, such long-distance transfers may impose compounded challenges in diet, microbiome, photoperiod and thermal regimes, with potential consequences for stress physiology and behavioural expression. Accordingly, selecting candidates based not only on genetic representation but also on behavioural plasticity and stress resilience may enhance acclimation and welfare, and could ultimately improve conservation outcomes. Future conservation planning would therefore benefit from longitudinal, repeated assessments of personality and physiological coping across transfers and ageing, enabling identification of phenotypes best suited to specific conservation roles, whether for reintroduction, managed relocation among breeding centres, or international programs.

While our study provides novel insights into the personality–cognition–physiology nexus in giant pandas, several limitations exist. First, the captive sample (*N* = 12) was male-biased and restricted to subadults/adults due to husbandry constraints. Although statistical controls accounted for this, the unbalanced structure precludes definitive conclusions regarding sex- or age-specific ontogenetic shifts. Second, although veterinary records confirmed females were not in oestrus or lactating during our July sampling (outside the peak February–May oestrus), future studies should explicitly quantify reproductive hormones alongside fGCM. Third, while potential seasonal variations in food motivation were mitigated by using highly preferred treats (e.g. carrots) and restricting each paradigm to a 2- to 3-month block, continuous longitudinal assessments are warranted. Finally, extrapolating from captive to wild contexts requires caution. The wild cohort was modest (*N* = 9), and the intra-individual fGCM–disturbance relationship, based on a single repeatedly sampled panda, remains preliminary. Larger wild datasets incorporating repeated measures and movement tracking are essential to robustly test the fitness consequences of these trait–hormone combinations *in situ*.

## Supplementary Material

Web_Material_coag019

## Data Availability

The data underlying this article are available in the article and its online supplementary material (Datasets S1–S3).
