# Supplementary material for: Panda profiles: integrating personality, cognition, and physiology for conservation success
Source: Conserv Physiol. 2026 Mar 27;14(1):coag019. doi: 10.1093/conphys/coag019 (PMC13034733; doi:10.1093/conphys/coag019)
Supplement: Web_Material_coag019 [file web_material_coag019.zip › APPENDIX_revised2.pdf]

APPENDIX SI

Methods

Hormone Extraction and Enzyme Immunoassay (EIA) Protocols

While the general workflow is described in the main text, specific laboratory parameters for reproducibility are detailed below:

a. Sample Preparation: Frozen faecal samples were lyophilised (freeze-dried) to eliminate water content variability. Dried samples were pulverised and sifted to remove coarse fibre.

b. Extraction: Hormones were extracted from 0.10 ± 0.01g of faecal powder using 5.0 mL of 90% ethanol. Samples were vortexed (30 min) and centrifuged (2500 rpm, 20 min). Supernatants were recovered and dried under forced air.

c. Assay Procedure:

   a) Coating: 96-well microtiter plates (Nunc-Immuno) were coated with Goat Anti-Rabbit IgG (Sigma R2004-5) and incubated at 4°C for 12–24 hours.

   b) Incubation: Reconstituted extracts (diluted 1:10 to 1:100 in assay buffer) were incubated with cortisol-HRP and cortisol antibody (R4866) at 37°C for 50 minutes.

   c) Colour Development: TMB substrate (Sigma T3405) was added, and plates were incubated at 37°C for approx. 30 minutes. The reaction was stopped with 2M H_2SO_4, and optical density was read at 450 nm (BioTek Elx800).

   d) Validation: Intra- and inter-assay coefficients of variation (CV) for high and low quality controls are reported in Table S1.

Table S1. Assay performance and quality-control (QC) characteristics for fGCM EIAs.

Assay	Plates (n)	Intra-assay CV (%)	Inter-assay CV (%)	QC High (%B)	QC High (pg/mL)	QC Low (%B)	QC Low (pg/mL)
fGCM	8	4.99 ± 3.46	6.05 ± 2.54	35.7 ± 1.1	2405.6 ± 242.6	74.3 ± 1.6	292.3 ± 33.2

Notes: Values are mean ± SD. QC samples (high/low) were in-house controls prepared from pooled panda faecal extracts and included on each plate. %B refers to percent bound.

Experiment 1: Multi-Access Box Test

Apparatus Details

The MAB utilised in our experiments was a square wooden structure measuring 840mm x 800 mm x 800 mm, featuring a base and a five-sided structure on top. The design was adapted from established paradigms for assessing physical cognition and innovation (Auersperg et al. 2011; Manrique et al. 2013; Huebner and Fichtel 2015; Johnson-Ulrich et al. 2018). The top surface was non-operable and designed with bars to facilitate external observation and scent dispersal. The four operable sides were equipped with distinct interactive mechanisms:

a. Side A (Push): A push flap (500 mm x 600 mm) opening inwardly.
b. Side B (Pull): A pull flap (500 mm x 600 mm) equipped with a grooved handle for outward opening.
c. Side C (Slide): Two sliding doors (each 250 mm x 600 mm) with grooved flanges, sliding laterally.
d. Side D (Drawer): A flush-bottom drawer (250 mm height) with a grooved handle.

Mechanisms could be mechanically blocked internally to control access according to the experimental phase.

Ethogram and Scoring Definitions

Detailed definitions of the behavioural metrics quantified are provided in Table S2. For Exploratory Diversity, the behavioural repertoire was quantified based on the occurrence of the following specific behaviours directed at the apparatus during the first exposure: sniffing, contacting with snout, biting, pushing with head, using paws to push/pull, climbing, embracing, standing on top, and moving through the box.

Behavioural assays

Behavioural measures were quantified using frame-by-frame video analysis. The specific operational definitions and ethogram used for scoring are detailed below:

1. Repeated Measures (Quantified per trial)

a. Persistence: Defined as the extent of engagement with the apparatus (Griffin and Guez 2014; van Horik et al. 2017). Calculated as the ratio of time spent actively exploring the MAB (physically manipulating or inspecting) to the total trial time.

b. Inhibitory Control (Inverse): Assessed by the inability to inhibit returning to a previously rewarded but currently blocked solution. Scored as the proportion of total exploration time spent interacting with blocked doors relative to the overall exploration time. A low inhibition score, indicating shorter time spent on blocked solutions, indicated superior inhibitory control.

c. Efficiency: Characterised by the latency from the start of the trial to the successful retrieval of food (Chow et al. 2016). This includes both active exploration and intervals of disengagement, capturing the total problem-solving time.

2. One-time Measures (Quantified during first exposure)

a. Neophobia: Measured as the latency (seconds) from the moment the subject first visually oriented towards the MAB to the moment of initial physical contact (Greggor et al. 2015).

b. Exploratory Diversity: A measure of the behavioural repertoire displayed towards the MAB during the first familiarisation trial. The score corresponds to the count of unique behaviours exhibited from the following ethogram (Griffin and Guez 2014; Benson-Amram et al. 2016; Chow et al. 2016):

   a) Sniffing: Olfactory investigation of any part of the box.
   b) Snout Contact: Touching the box directly with the nose/snout without biting.
   c) Biting: Using teeth to gnaw or pull on parts of the box.
   d) Head Push: Using the forehead or side of the head to apply force.
   e) Paw Manipulation: Using forepaws to push, pull, or scratch surfaces.
   f) Climbing: ascending onto the box with all four limbs off the ground.
   g) Embracing: Wrapping forelimbs around the box.
   h) Standing On: Standing on the top surface of the box.
   i) Moving Through: Physically entering or passing through an open compartment.

Table S2 Summary of behavioural measures used in MAB test.

	Term	Definition
	Measures extracted once per trial	
1	Persistence	Ratio of time spent exploring the MAB to total trial time
2	Efficiency	The time elapsed from the start of the trial to the successful retrieval of food
3	Inhibitory Control	Amount of time spent in exploration with blocked solutions relative to total exploration time
4	Exploration Duration*	Duration of time subjects spent in exploring MAB
	Measures extracted once per subject	
5	Neophobia	Latency from trial start to contact with MAB on first familiarization trial
6	Exploratory Diversity	Variety of behaviours displayed towards MAB on first familiarization trial
7	Total Experiments Completed	The total number of trials conducted by the subject

*Exploration duration was only used in to assess learning

Experiment 2: Resource Depletion Test

Apparatus Details

The apparatus consisted of six identical opaque paper bowls (12 cm diameter x 6.8 cm height) arranged in a fixed semi-circular configuration within the 3m x 3 m testing enclosure. The spatial arrangement of the bowls relative to the enclosure landmarks remained constant across all phases and trials.

Ethogram and Scoring Definitions

Detailed definitions of the behavioural metrics quantified are provided in Table S3.

Table S3 Summary of behavioural measures used in Resource Depletion Test.

	Term	Definition
	Fixed Resource Phase	
1	Pick Count	The number of attempts a subject needs to find the food during each trial.
2	Trials to Learn*	The number of trials required for a subject to consistently find food with no more than two picks in three consecutive trials.
	Resource Depletion Phase	
3	Delayed Fixation Trials (DFT)*	The Number of trials required for a subject to not return to the initially learned feeding point until at least three picks in three consecutive trials.
4	Learned Location Visit Ratio (LLVR)	$\mathrm{LLVR} = \frac{\mathrm{Visits}_{\mathrm{learned}}}{\mathrm{Visits}_{\mathrm{total}}}$
5	Learned Location Stay Duration (LLSD)	$\mathrm{LLSD} = \frac{\mathrm{Time}_{\mathrm{learned}}}{\mathrm{Time}_{\mathrm{total}}}$
6	Exploration Accuracy	$\mathrm{Accuracy} = 1 - \frac{\mathrm{Picks}_{\mathrm{unsuccessful}}}{\mathrm{Picks}_{\mathrm{total}}}$
7	Working Memory Errors (WME)	Revisits to any location within the same trial, identifying repeated explorations of the same point.
8	Total Locations Visited (TLV)	Total number of locations visited during the trials.

*Indicates metrics that quantified only once

Experiment 3: String-Pulling Tasks

Apparatus Details

The task utilised braided hemp ropes with a diameter of 14 mm. Ropes were arranged on the floor of the 3m x 3m testing room. The diameter was selected to ensure easy grasping and manipulation by giant pandas.

Quantification of Lateralisation

Hand preference was recorded for every trial. To quantify the direction and strength of lateralisation, we calculated two indices:

a. Handedness Index (HI): Calculated as HI = (R - L) / (R + L), where R and L represent the total number of right and left hand uses, respectively. HI values range from -1.0 (exclusive left use) to +1.0 (exclusive right use). The absolute value (|HI|) indicates the strength of the preference.

b. Binomial Z-score: Used to assess the statistical significance of the preference. Calculated as:

$$Z = \frac{R - 0.5 \times N}{\sqrt{0.25 \times N}}$$

where N is the total number of trials (R + L). Individuals with $|Z| > 1.96$ were classified as having a significantly lateralised hand preference ($p < 0.05$).

Field Study of Wild Giant Pandas

1. **Field Sampling Protocol**

Faecal freshness was assessed visually according to the rubric by Deng et al. (2014) to minimise hormone degradation. Only samples classified as <7 days old were collected:

a. 1 day or less (mucous present on both surfaces, appearing fresh and lustrous);
b. 1-3 days (diminished mucous lustre on the upper surface, lower still shiny);
c. 3-7 days (lustre lost on the upper surface, some retained on the lower);
d. 7-15 days (no mucous lustre on the upper, lower surface with spider silk or hyphae);
e. over 15 days (feces that have lost the smell of bamboo, appear mildewed, or are dry and decayed)

Samples were differentiated into two subsamples:

a. DNA Subsample: External mucous layer (rich in intestinal epithelial cells) preserved in 99% ethanol.
b. Hormone Subsample: Core faecal matter, sealed in zip-lock bags and frozen at −20°C within 12 hours.

2. DNA Extraction and Species Verification

Genomic DNA was extracted using the TIANamp Stool DNA Kit (Tiangen Biotech, Beijing) following the manufacturer's protocol. Species identity was verified by amplifying the mitochondrial control region using primers P-tp (5'-CTCCCTAAGACTCAAGGAAG-3') and BEDH (5'-GGGTGATCTATAGTGTTATGTCC-3').

a. PCR Conditions: 20μL reaction volume containing 0.5μL each of 10μM forward/reverse primers, 3μL DNA template, 10μL 2x M5 HiPer Plus Taq HiFi PCR Mix, and 6μL ddH_2O.
b. Cycling Parameters: Initial denaturation at 95°C (3 min); 40 cycles of [94°C (25 s), 55°C (25 s), 72°C (15 s)]; final extension at 72°C (5 min).
c. Verification: Products were visualised on 1.5% agarose gel. Only samples yielding a single ~750 bp band were retained for downstream genotyping.

3. Microsatellite Genotyping

Individual identification utilised 8 tetranucleotide microsatellite loci (Standard: DB51/T2404-2017; primers listed in Table S4).

a. PCR Amplification: Performed in an S1000™ Thermal Cycler. Protocol: 94°C (5 min); 40 cycles of [94°C (50 s), locus-specific annealing temperature (60–63°C) (45 s), 72°C (30 s)]; final extension at 72°C (10 min).

b. Fragment Analysis: PCR products were separated on an ABI 3730 DNA Analyser with internal standard GS500LIZ. Alleles were scored using GeneMapper v4.0.

c. Quality Control (QC): To ensure reliability, a "multi-tube" approach was used (Taberlet et al. 1996):

    a) Homozygotes: Confirmed by observation in ≥ 3 independent positive PCRs.

    b) Heterozygotes: Each allele confirmed in ≥ 2 independent PCRs.

    c) Genotyping Errors: Micro-Checker (Van Oosterhout et al. 2004) was used to test for null alleles, large allele dropout, and stuttering. Samples failing to produce consensus genotypes after 4 attempts were discarded.

4. Individual Identification and Genetic Diversity

Individual identity analysis was performed in CERVUS 3.0 (Gutiérrez-Espeleta et al. 2000). The discriminatory power of the 8-locus marker set was validated using GIMLET (Valière 2002). The probability of identity for siblings ($P_{ID(sib)}$) was required to be <0.01 to confidently distinguish individuals.

Statistical Verification and Criteria

1. PCA Suitability and Retention Criteria

Principal Component Analysis (PCA) was only conducted when the correlation matrix satisfied the following statistical requirements:

a. Correlation Strength: Variable sets exhibited high intercorrelations ($|r| > 0.7$).

b. Sampling Adequacy (KMO): The Kaiser-Meyer-Olkin measure of sampling adequacy required a value ≥ 0.50 (Dziuban and Shirkey 1974; Dziuban et al. 1979; Fouladi and Steiger 1993; Budaev 2010).

c. Sphericity (Bartlett): Bartlett's Test of Sphericity required significance ($p < 0.05$) to ensure the correlation matrix was not an identity matrix (Fouladi and Steiger 1993).

d. Component Retention: The number of retained components was determined using the Kaiser criterion (eigenvalues > 1) and visual inspection of the scree plot (Bates et al. 2015; Greggor et al. 2015).

2. Covariate Selection Strategy

To avoid over-parameterizing the GLMs with unnecessary control variables, potential covariates (age class, sex, body weight) were pre-screened for each behavioural metric:

a. Categorical predictors (Age, Sex): Assessed using Kruskal-Wallis tests.

b. Continuous predictors (Body Weight): Assessed using linear regression. Only covariates showing a significant association ($p < 0.05$) with the behavioural response variable were retained as fixed effects in the final GLMs.

3. Variable Grouping for PCA (Experiment 2)

For the Resource Depletion Test, behavioural metrics were theoretically categorised prior to dimension reduction to ensure interpretability:

a. Learning Performance Metrics: Pick Count, Trials to Learn, Delayed Fixation Trials (DFT), and Working Memory Errors (WME).

b. Exploratory/Personality Metrics: Learned Location Visit Ratio (LLVR), Learned Location Stay Duration (LLSD), Exploration Accuracy, and Total Locations Visited (TLV).

Separate PCAs were conducted for these two clusters to independently derive composite scores for "Learning Capability" and "Exploratory Style."

4. Cross-Experiment Analytical Strategy

To explore the integration of traits across different cognitive domains (Experiments 1–3), we first conducted a comprehensive cross-correlation analysis of all derived metrics. To minimize Type I errors from multiple comparisons, Generalized Linear Models (GLMs) linking traits across experiments were only constructed for metric pairs exhibiting a moderate-to-strong correlation (Spearman's $|\rho| > 0.5$).

Table S4. Characteristics of 8 Tetranucleotide Microsatellite Loci

Microsatellite	Core Sequence	Primer Sequences	Annealing Temperature (°C)
GPL60	(TCTT)12	F:TGCCGGAAAGTTCTAAGCAT<br>R: TTTCTCTCCCTCTCCCCTTC	63
GPL8	(ATCC)11	F:TGGTTTTGCAAGGATGACAG<br>R:TTGTGACAAGCAAGCTCCAC	63
GPL29	(ATCC)19	F: TCCAAGGCTTCAAACAAGGT<br>R: CACCACAGGTGCCAATTATG	60
gpz20	(AAAG)10	F: CCCTCTCGTTGTGTCTCTCTG<br>R: CACCTGGTAAATGGCACCTT	63
gpz47	(AATG)20	F: GACCTCAGTGTACGCCCAGT<br>R: CTGGACAGGCAGGTAGAAGC	60
gpy5	(AACT)16	F: CTCGGGAGCTTTGTACCATC<br>R: CAGAGAGCCCAAACCTCAAC	63
gpz6	(AAAG)11	F: CCTGGCAGGGCAAAGTATT<br>R: CCCCGTGAAAACATCAAGAC	60
gpy20	(TTTG)16	F: GCAGGCACTCAAGAGGTGTT<br>R:CCTTGTGCTAAACACAGGTGA	63

*All primers are labelled with FAM.

Table S5. Individual raw fGCM concentrations (ng/g) collected over the 5-day standardised sampling window.

Subject Name	Sample 1	Sample 2	Sample 3	Sample 4	Sample 5	Mean
**E Mei**	145.5	699	352.5	418.8	596.7	442.5
**Lin Xi**	347.3	771.4	404.6	495.4	351.8	474.1
**Mao Mao**	215.9	328.6	409.5	358	200.4	302.5
**Mu Ye**	445.8	1130.6	211.3	302.5	355.9	489.2
**Nong Nong**	409.8	376.2	351.6	566.2	347.4	410.2
**Qing Qing**	280.3	256.4	250.3	362.6	482.5	326.4
**Su Yang**	243.3	121.8	235.8	105.7	123.1	165.9
**Xing Ye**	868.6	83.7	95.4	149.1	85.8	256.5
**Xing Yue**	281.5	264.8	548.7	255.8	259	322
**Yun Yun**	212.7	139.2	70.6	268.6	143.1	166.8
**Zhu Ling**	136.5	249.2	103.9	106.4	341.4	187.5
**Ling Lang**	536.2	560.5	487.3	433.3	379.4	479.3

Table S6. Microsatellite Genotype Data at Eight Loci for Wild Giant Panda Samples

Samples	GPL8a	GPL8b	GPL29a	GPL29b	GPL60a	GPL60b	gpz20a	gpz20b	gpz47a	gpz47b	gpy5a	gpy5b	gpy20a	gpy20b	gpz6a	gpz6b
Q-D-9	0	0	159	159	225	225	266	282	174	186	204	204	149	149	0	0
Q-D-10	228	228	159	159	221	221	266	266	174	198	0	0	0	0	194	210
Q-D-11	232	232	159	159	221	221	266	266	178	198	0	0	0	0	0	0
Q-D-12	0	0	0	0	217	217	298	298	190	198	212	212	149	149	0	0
Q-D-13	0	0	0	0	217	217	286	286	178	190	0	0	149	149	198	202
Q-D-14	0	0	159	159	225	225	278	278	174	178	204	204	149	149	0	0
Q-D-15	228	228	159	159	225	225	266	286	174	186	0	0	149	149	214	214
Q-D-16	0	0	163	163	213	221	258	258	182	198	208	208	149	149	198	198
Q-D-17	224	236	159	159	225	225	278	278	178	182	200	200	0	0	222	222
Q-D-18	0	0	163	163	221	221	0	0	186	198	208	208	0	0	214	214
Q-D-20	0	0	171	171	225	225	282	294	174	198	0	0	0	0	218	218
Q-D-21	236	236	0	0	221	221	290	290	174	198	0	0	0	0	202	210

Q-D-25	236	236	163	163	0	0	290	290	174	198	208	208	149	149	210	210
Q-D-26	0	0	163	163	0	0	0	0	178	198	208	208	149	149	206	206
Q-D-27	224	224	0	0	221	221	274	274	174	198	0	0	165	165	206	206
Q-D-29	0	0	163	163	221	221	0	0	186	198	212	212	0	0	194	210
Q-D-30	0	0	0	0	233	233	298	298	198	202	200	200	149	149	206	206
Q-D-32	236	236	0	0	237	237	0	0	182	182	212	212	149	149	0	0

Table S7. Intra-individual repeatability of repeated behavioural metrics in the Resource Depletion Test.

Behavioural metric	χ^2	p	ICC	95% CI
Pick count	13.814	<0.001	0.168	0.007–0.352
Learned-location visit ratio (LLVR)	35.325	<0.001	0.3	0.072–0.650
Learned-location stay duration (LLSD)	4.067	0.05	0.081	0.007–0.247
Exploration accuracy	4.08	0.05	0.081	0.000–0.227
Working memory errors (WME)	18.32	<0.001	0.196	0.034–0.434
Total locations visited (TLV)	34.662	<0.001	0.292	0.052–0.524

Repeatability was estimated as intraclass correlation coefficients (ICC) from linear mixed-effects models with individual identity as a random intercept. Significance of the random effect was assessed using likelihood ratio tests (χ^2). 95% confidence intervals (CI) were obtained by bootstrap.

Table S8. Effects of demographic background factors on behavioural/personality metrics across captive experiments.

Metric	Sex (H)	Sex (p)	Age class (H)	Age class (p)	Body mass (β)	Body mass (p)
**Multi-Access Box (MAB) test**						
Reactive score	0.042	0.838	0.667	0.414	0.002	0.939
Exploratory diversity	0.045	0.832	0.719	0.396	−0.003	0.888
**Resource Depletion test**						
Learning impediment score	1.846	0.174	0.692	0.405	−0.004	0.864
Exploratory adaptiveness score	1.038	0.308	3.085	0.079	0.032	0.109
Learned-location visit ratio (LLVR)	0.891	0.345	0.428	0.513	0	0.88
Working memory errors (WME)	0.876	0.349	0.695	0.405	−0.011	0.473
Total locations visited (TLV)	0.462	0.497	1.034	0.309	−0.025	0.329
**String-Pulling Tasks**						
DRS (Parallel Phase)	0.029	0.864	0.702	0.402	-0.319	0.649
DRS (Cross Phase)	0.26	0.61	3.085	0.079	0.14	0.844

Sex and age class were tested using Kruskal–Wallis tests (H). Body mass was tested using simple linear regression (β). None of the covariates showed significant effects (all $p > 0.05$).

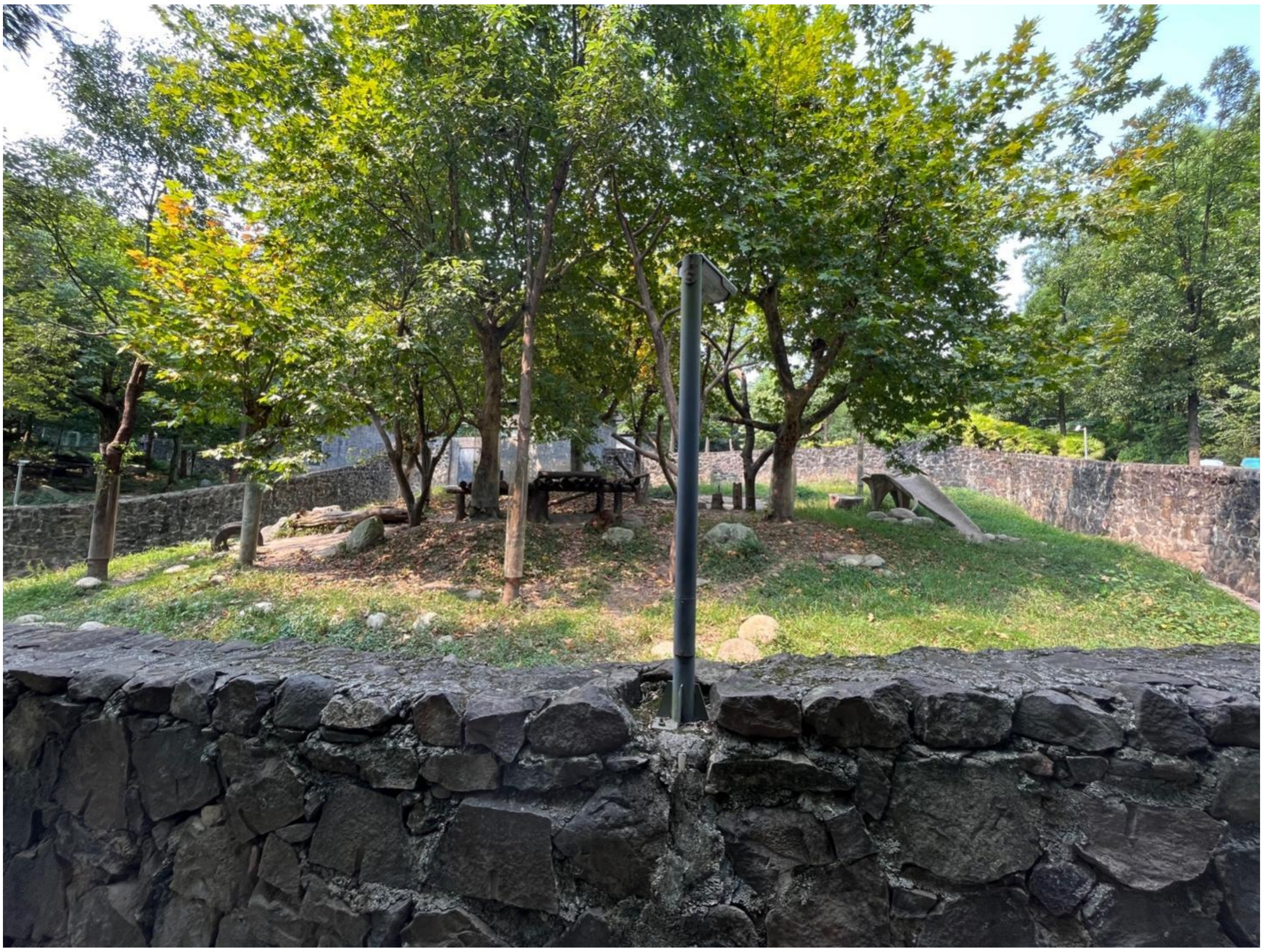

Supplementary Figure 1. The environment of the outer enclosure of pandas.

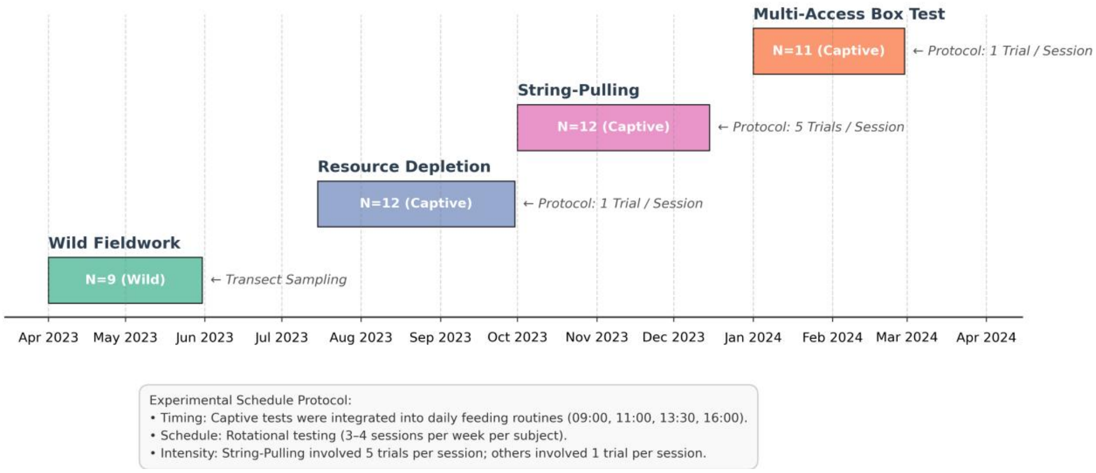

Supplementary Figure 2. Experimental timeline and detailed testing protocol. The study was conducted from April 2023 to February 2024. (A) Green bar represents fieldwork on the wild population (N=9) in 2023. (B) Blue, purple, and orange bars represent the sequential captive experiments (N=12 or 11). Captive testing was strictly integrated into the four daily feeding routines to minimise stress. A rotational schedule was employed (Monday–Friday), resulting in a frequency of 3–4 testing sessions per subject per week.

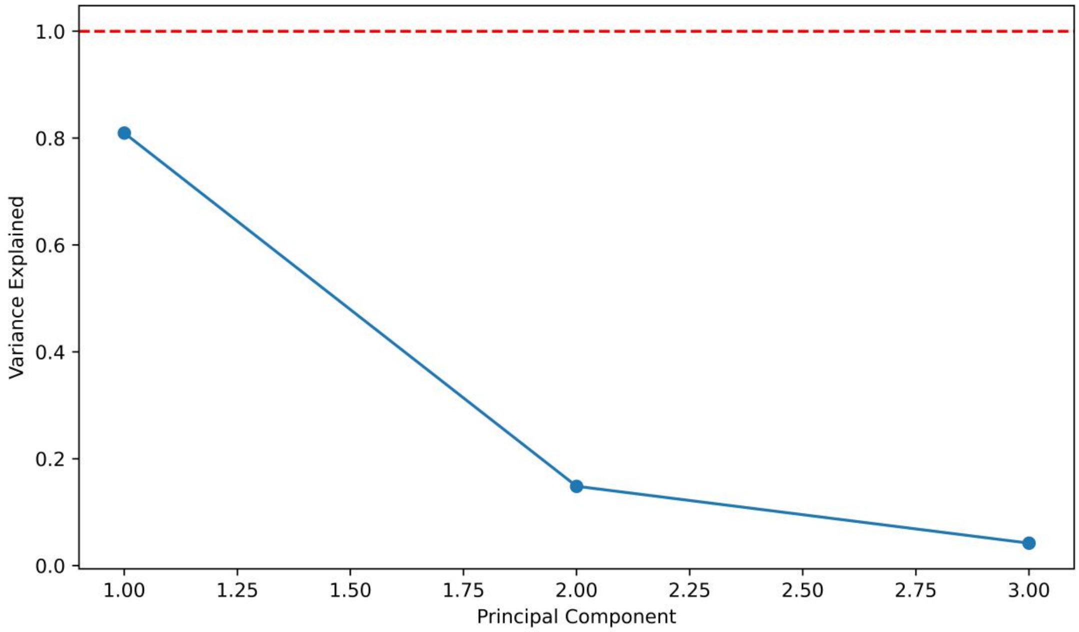

Supplementary Figure 3. Scree plot of behavioural traits in MAB.

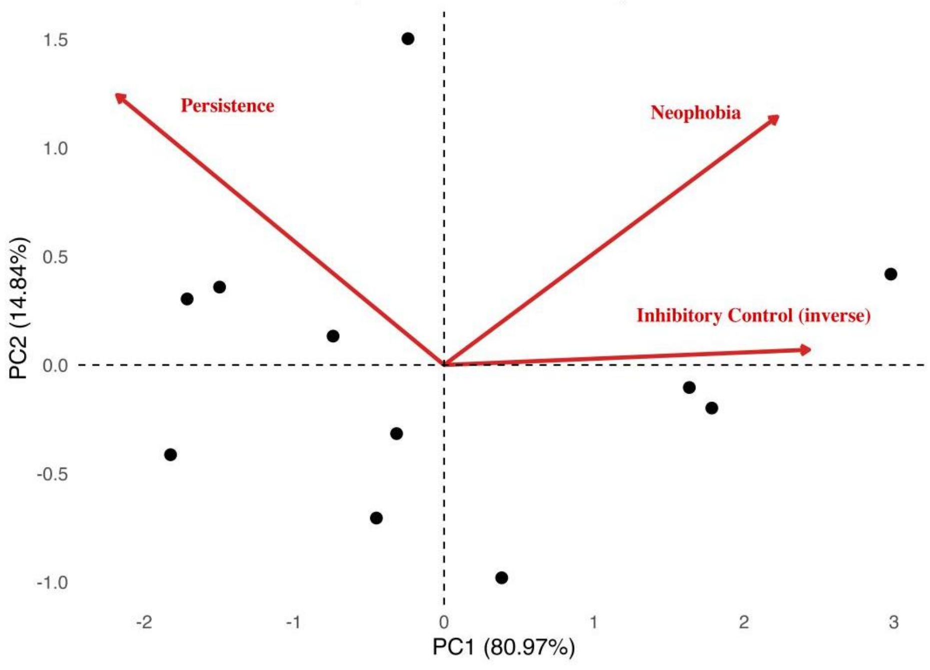

Supplementary Figure 4. PCA of behavioural traits in MAB.

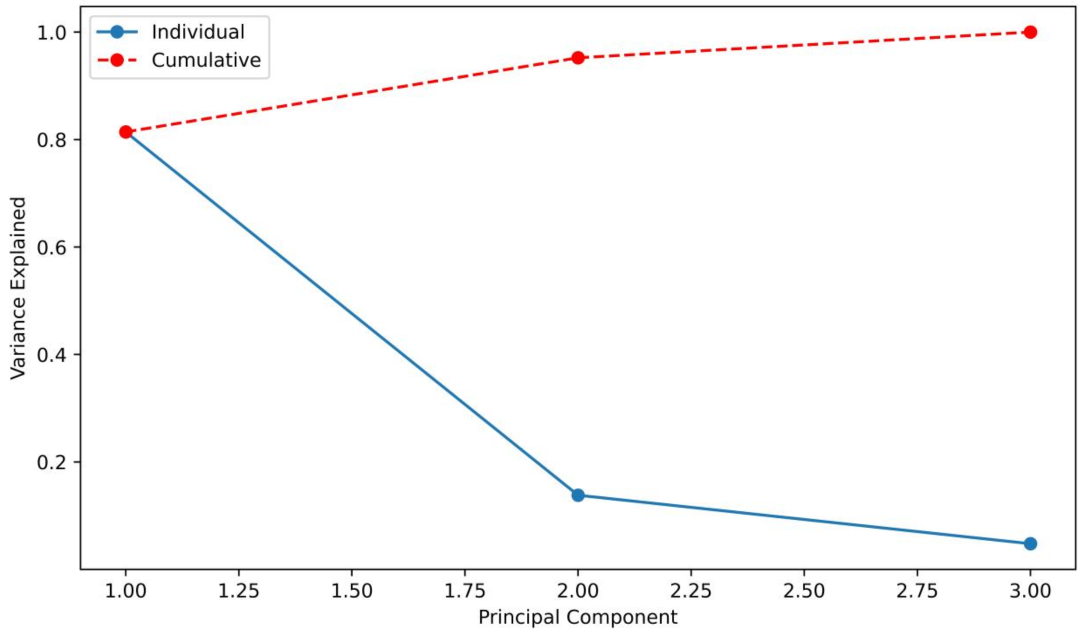

Supplementary Figure 5. Scree plot of personality metrics in Resource Depletion Test.

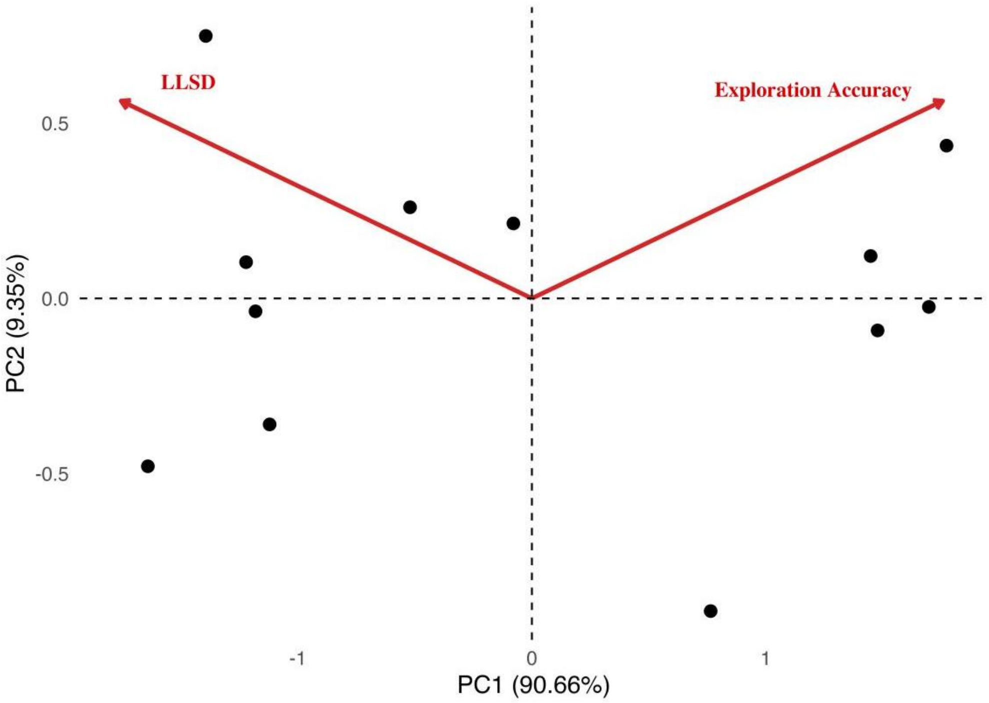

Supplementary Figure 6. PCA of personality metrics in Resource Depletion Test.
*LLSD: Learned Location Stay Duration, Proportion of time spent at the learned location against the overall exploration time.

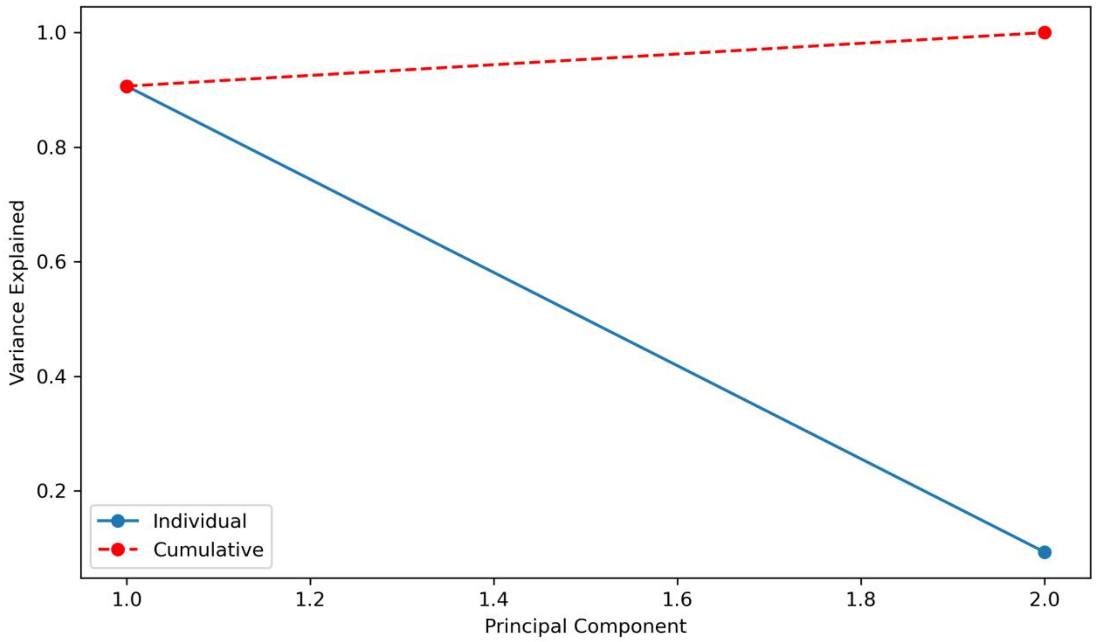

Supplementary Figure 7. Scree plot of learning metrics in Resource Depletion Test.

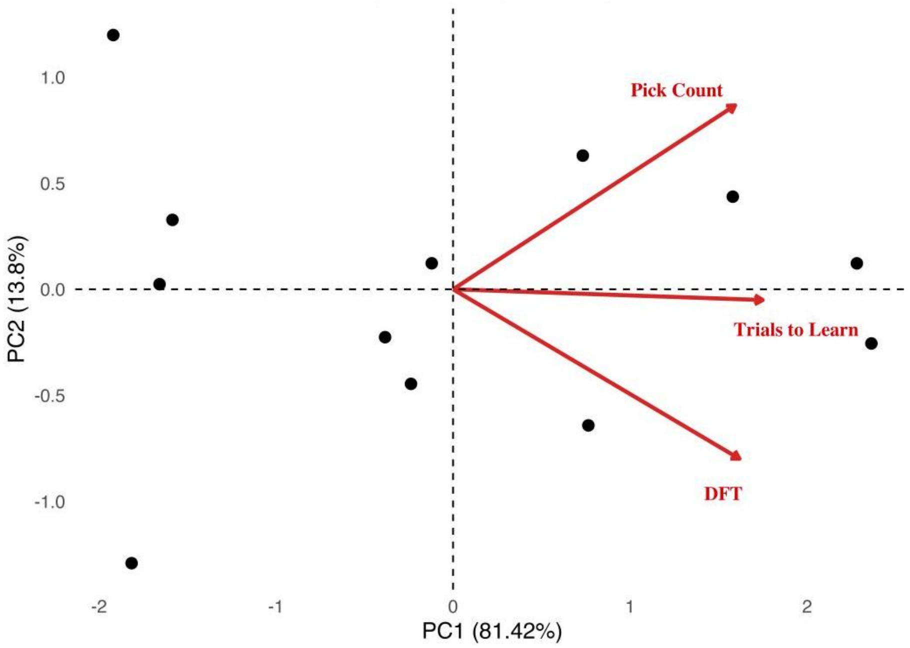

Supplementary Figure 8. PCA of learning metrics in Resource Depletion Test. *DFT: Delayed Fixation Trials, the Number of trials required for a subject to not return to the initially learned feeding point until at least three picks in three consecutive trials.

Movie legends

Video S1. This video demonstrates the decision-making process of a giant panda during a string-pulling task. The panda carefully evaluates the options before making a clear and deliberate choice to pull the string.

Auersperg, A. M., Von Bayern, A. M., Gajdon, G. K., Huber, L., Kacelnik, A., 2011. Flexibility in problem solving and tool use of kea and New Caledonian crows in a multi access box paradigm. PLoS One 6, e20231.

Bates, D., Mächler, M., Bolker, B., Walker, S., 2015. Fitting Linear Mixed-Effects Models Using lme4. Journal of Statistical Software 67, 1 - 48.

Benson-Amram, S., Dantzer, B., Stricker, G., Swanson, E. M., Holekamp, K. E., 2016. Brain size predicts problem-solving ability in mammalian carnivores. Proceedings of the National Academy of Sciences 113, 2532-2537.

Budaev, S. V., 2010. Using Principal Components and Factor Analysis in Animal Behaviour Research: Caveats and Guidelines. Ethology 116, 472-480.

Chow, P. K. Y., Lea, S. E. G., Leaver, L. A., 2016. How practice makes perfect: the role of persistence, flexibility and learning in problem-solving efficiency. Animal Behaviour 112, 273-283.

Deng, H., Jin, X., Hu, D., Liu, D., 2014. Fecal cortisol content of wild giant pandas (*Ailuropoda melanoleuca*) to monitor human disturbance level in natural habitats. Animal Biology 64, 75-86.

Dziuban, C. D., Shirkey, E. C., 1974. When is a correlation matrix appropriate for factor analysis? Some decision rules. Psychological bulletin 81, 358.

Dziuban, C. D., Shirkey, E. C., Peeples, T. O., 1979. An investigation of some distributional characteristics of the measure of sampling adequacy. Educational and Psychological Measurement 39, 543-549.

Fouladi, R. T., Steiger, J. H., 1993. Tests of Multivariate Independence: A Critical Analysis of" A Monte Carlo Study of Testing the Significance of Correlation Matrices" by Silver and Dunlap. Educational and Psychological Measurement 53, 927-932.

Greggor, A. L., Thornton, A., Clayton, N. S., 2015. Neophobia is not only avoidance: improving neophobia tests by combining cognition and ecology. Current Opinion in Behavioral Sciences 6, 82-89.

Griffin, A. S., Guez, D., 2014. Innovation and problem solving: a review of common mechanisms. Behavioural Processes 109, 121-134.

Gutiérrez-Espeleta, G. A., Kalinowski, S. T., Boyce, W. M., Hedrick, P. W., 2000. Genetic variation and population structure in desert bighorn sheep: implications for conservation. Conservation Genetics 1, 3-15.

Huebner, F., Fichtel, C., 2015. Innovation and behavioral flexibility in wild redfronted lemurs (*Eulemur rufifrons*). Animal Cognition 18, 777-787.

Johnson-Ulrich, L., Johnson-Ulrich, Z., Holekamp, K., 2018. Proactive behavior, but not inhibitory control, predicts repeated innovation by spotted hyenas tested with a multi-access box. Animal Cognition 21, 379-392.

Manrique, H. M., Völter, C. J., Call, J., 2013. Repeated innovation in great apes. Animal Behaviour 85, 195-202.

Taberlet, P., Griffin, S., Goossens, B., Questiau, S., Manceau, V., Escaravage, N., Waits, L. P., Bouvet, J., 1996. Reliable genotyping of samples with very low DNA quantities using PCR. Nucleic acids research 24, 3189-3194.

Valière, N., 2002. GIMLET: a computer program for analysing genetic individual identification data. Molecular Ecology Notes 2, 377-379.
Van Horik, J. O., Langley, E. J., Whiteside, M. A., Madden, J. R., 2017. Differential participation in cognitive tests is driven by personality, sex, body condition and experience. Behavioural Processes 134, 22-30.
Van Oosterhout, C., Hutchinson, W. F., Wills, D. P. M., Shipley, P., 2004. micro-checker: software for identifying and correcting genotyping errors in microsatellite data. Molecular Ecology Notes 4, 535-538.
